# Supplementary material for: Neural signaling contributes to heart formation and growth in the invertebrate chordate, Ciona robusta
Source: bioRxiv. 2025 May 2:2025.04.28.651085. Preprint. [Version 1] doi: 10.1101/2025.04.28.651085 (PMC12247680; doi:10.1101/2025.04.28.651085)
Supplement: 1 [file NIHPP2025.04.28.651085V1-supplement-1.pdf]

bioRxiv preprint doi: https://doi.org/10.1101/2025.04.28.651085; this version posted May 2, 2025. The copyright holder for this preprint (which was not certified by peer review) is the author/funder, who has granted bioRxiv a license to display the preprint in perpetuity. It is made available under aCC-BY 4.0 International license.

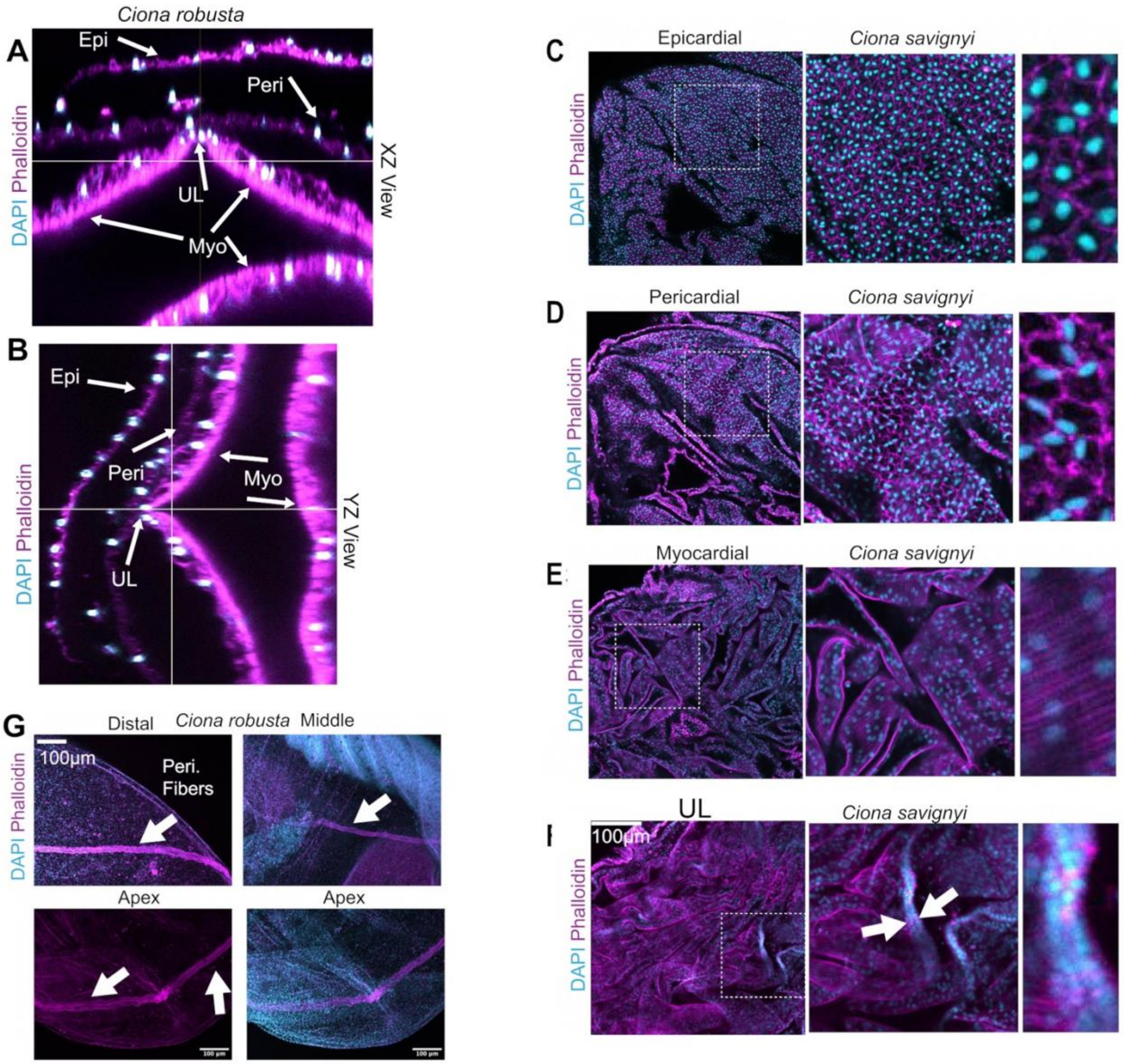

S1 Fig. Microanatomy of *C. robusta* and *C. savignyi* adult hearts. (A) Cross-section (X-Y) view of adult *C. robusta* heart. **(B)** Cross-section (Y-Z) view of *C. robusta* adult heart. **(C-F)** *C.*

bioRxiv preprint doi: https://doi.org/10.1101/2025.04.28.651085; this version posted May 2, 2025. The copyright holder for this preprint (which was not certified by peer review) is the author/funder, who has granted bioRxiv a license to display the preprint in perpetuity. It is made available under aCC-BY 4.0 International license.

savignyi cell types as labeled, arrows in (**F**) indicate the UL. Middle panels are magnified areas from the left panels, right panels are magnified areas from middle panels. **(G)** Previously unidentified ordered pericardial, F-actin-rich fascicles spanning the heart from posterior (distal) to anterior (apex) on the pericardial layer. These fibers were only prominent in young adult *C. robusta* hearts. In all images cyan represents DAPI staining and magenta represents phalloidin staining.

bioRxiv preprint doi: https://doi.org/10.1101/2025.04.28.651085; this version posted May 2, 2025. The copyright holder for this preprint (which was not certified by peer review) is the author/funder, who has granted bioRxiv a license to display the preprint in perpetuity. It is made available under aCC-BY 4.0 International license.

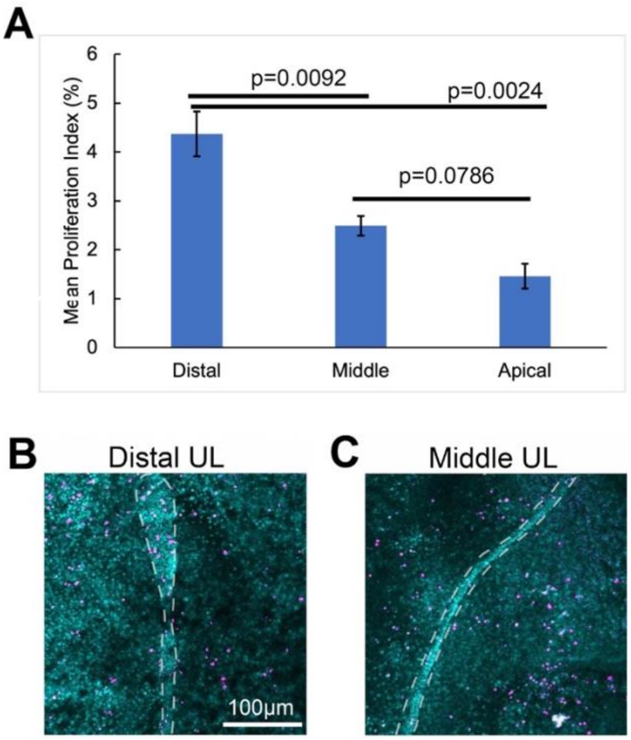

S2 Fig. Regional cell division frequency in the adult heart. (A) Graph displaying mean proliferation index (% dividing cells) in distal, middle and apical regions of the heart as assayed by 24-hour pulses of EdU, N=15 adult hearts. Error bars represent the S.E.M., and p-value calculated from t-test. **(B-C)** Representative images of distal and midline UL proliferation respectively as assayed by a 6-hour pulse of EdU (magenta), DAPI stained nuclei (cyan).

bioRxiv preprint doi: https://doi.org/10.1101/2025.04.28.651085; this version posted May 2, 2025. The copyright holder for this preprint (which was not certified by peer review) is the author/funder, who has granted bioRxiv a license to display the preprint in perpetuity. It is made available under aCC-BY 4.0 International license.

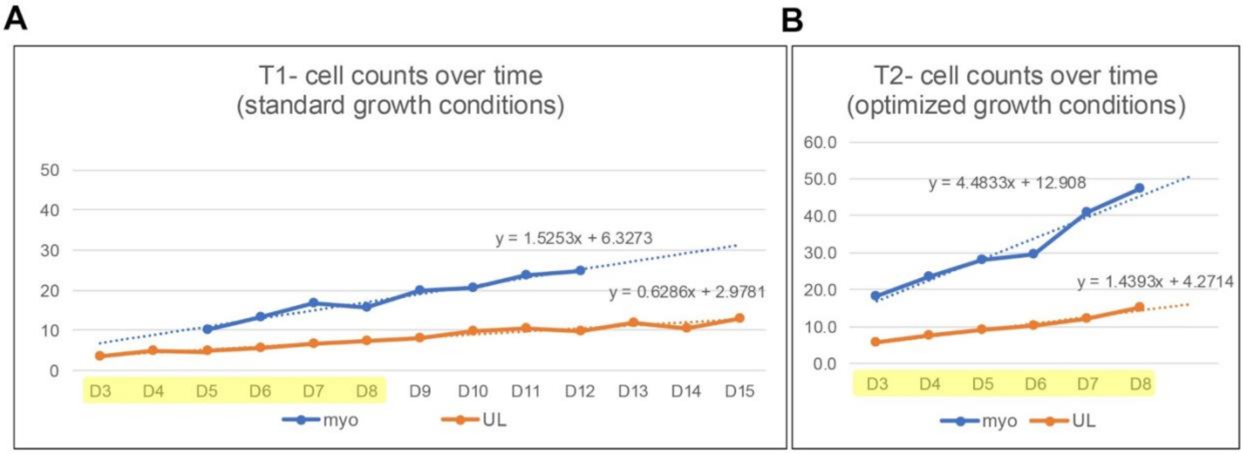

S3 Fig. Juvenile midline UL and myocardial growth rates. (A) Quantity of myocyte (blue)

bioRxiv preprint doi: https://doi.org/10.1101/2025.04.28.651085; this version posted May 2, 2025. The copyright holder for this preprint (which was not certified by peer review) is the author/funder, who has granted bioRxiv a license to display the preprint in perpetuity. It is made available under aCC-BY 4.0 International license.

and midline UL cells (orange) from D3 to D15 using standard culturing conditions. **(B)** Quantity of myocyte (blue) and midline UL cells (orange) from D3 to D8 days using optimized culturing conditions (see Methods section). In A and B, D3-D8 along the x-axis are highlighted for comparison. Orange dots and lines, number of UL cells per time point.

bioRxiv preprint doi: https://doi.org/10.1101/2025.04.28.651085; this version posted May 2, 2025. The copyright holder for this preprint (which was not certified by peer review) is the author/funder, who has granted bioRxiv a license to display the preprint in perpetuity. It is made available under aCC-BY 4.0 International license.

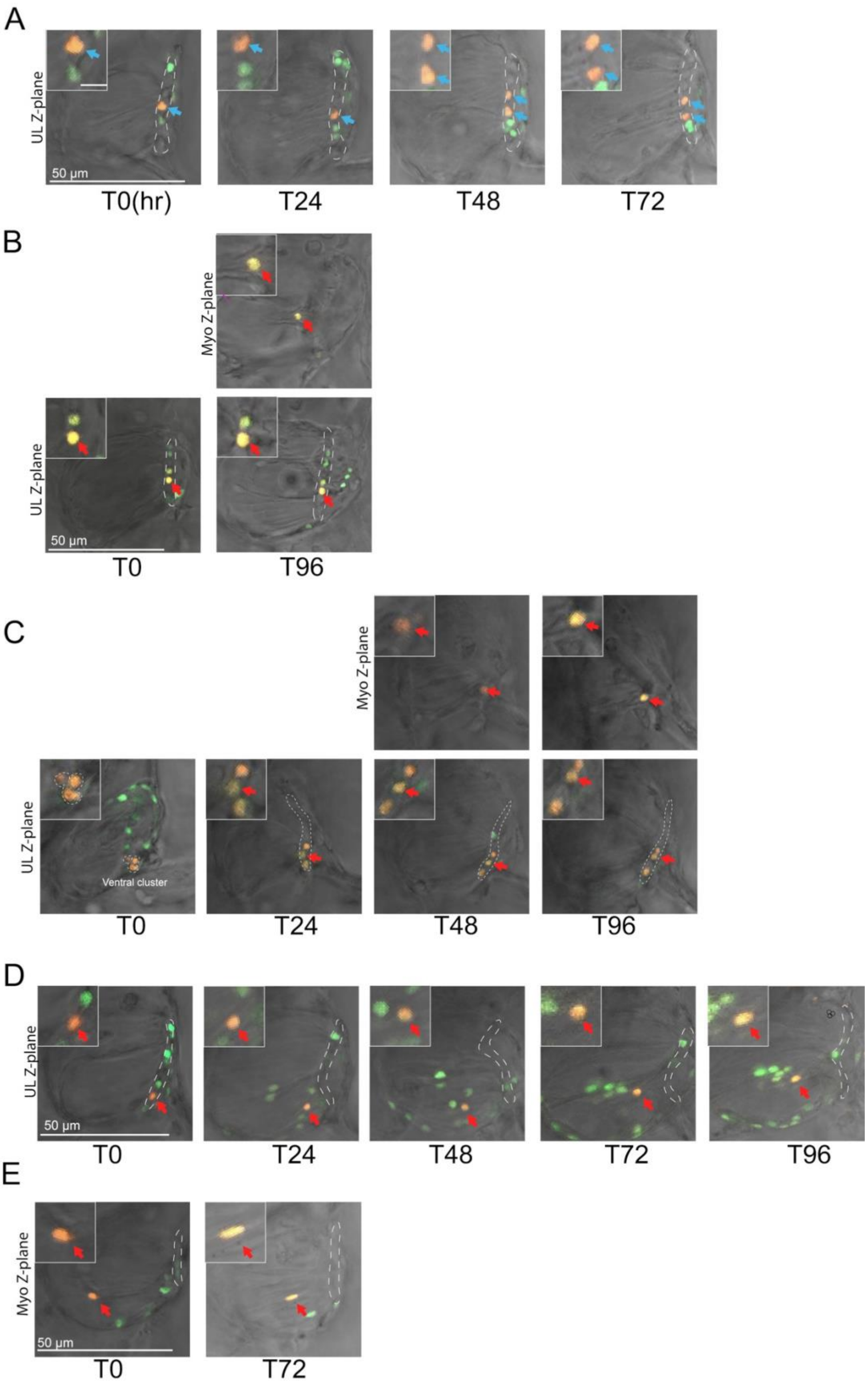

417

bioRxiv preprint doi: https://doi.org/10.1101/2025.04.28.651085; this version posted May 2, 2025. The copyright holder for this preprint (which was not certified by peer review) is the author/funder, who has granted bioRxiv a license to display the preprint in perpetuity. It is made available under aCC-BY 4.0 International license.

S4 Fig. Lineage tracing of myocardial progenitors in the UL using Kaede conversion. In all panels, photoconverted cells (yellow) are tracked over multiple days in living juveniles starting with photoconversion at D5, timepoints indicate hours after conversion. See text for details. **(A)** Blue arrows track a single labeled midline UL cell that appears to divide symmetrically to produce two midline UL daughters. **(B, C)** Red arrows track single labeled midline UL cells that appears to divide asymmetrically to produce a midline UL daughter that remains in the same confocal plane as the rest of the UL, bottom row, along with a presumptive myocardial precursor that moves into a different confocal plane (top row). **(D)** Red arrows track a single labeled presumptive myocardial precursor that appears to migrate anteriorly, away from the UL. **(E)** Red arrows track a single labeled presumptive myocardial precursor that appears to mature over a 72-hr time-course as evidenced by elongation of the nucleus.

bioRxiv preprint doi: https://doi.org/10.1101/2025.04.28.651085; this version posted May 2, 2025. The copyright holder for this preprint (which was not certified by peer review) is the author/funder, who has granted bioRxiv a license to display the preprint in perpetuity. It is made available under aCC-BY 4.0 International license.

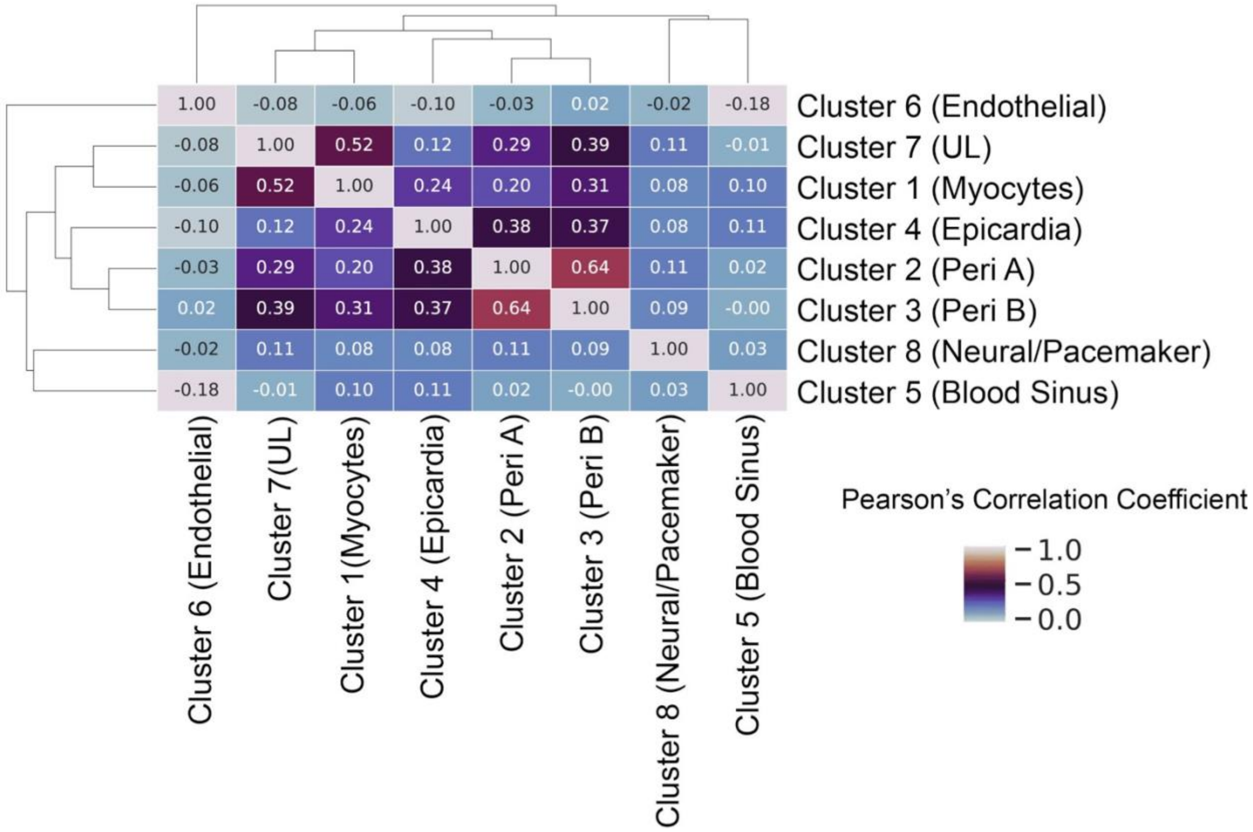

S5 Figure. Pearson's correlation coefficient (PCC) analysis to assess cluster similarities. PCC suggests Cluster 2 correlates strongly with Cluster 3 while Cluster 1 correlates strongly with Cluster 7. Overlapping expression patterns between these two clusters may contribute to

bioRxiv preprint doi: https://doi.org/10.1101/2025.04.28.651085; this version posted May 2, 2025. The copyright holder for this preprint (which was not certified by peer review) is the author/funder, who has granted bioRxiv a license to display the preprint in perpetuity. It is made available under aCC-BY 4.0 International license.

the lack of uniquely enriched genes in Cluster 1 (Main text Figure 3A, B). Additionally, PCC revealed Cluster 8 did not correlate strongly with any other cluster suggesting this cluster is transcriptionally unique.

bioRxiv preprint doi: https://doi.org/10.1101/2025.04.28.651085; this version posted May 2, 2025. The copyright holder for this preprint (which was not certified by peer review) is the author/funder, who has granted bioRxiv a license to display the preprint in perpetuity. It is made available under aCC-BY 4.0 International license.

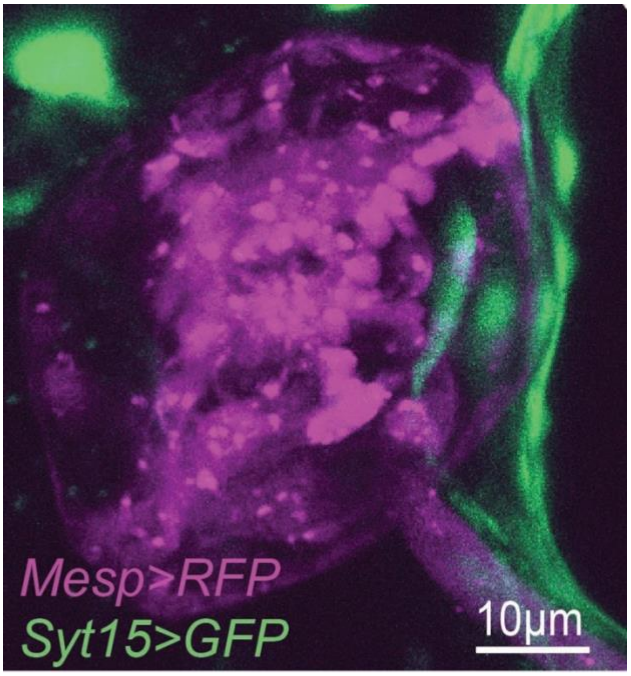

S6 Figure. *Synaptotagmin 15* reporter expression in the epicardium of a D15 juvenile.
Mesp>RFP labels pericardium, myocardium, and the UL (magenta). *Syt15>GFP* detected in the overlying epicardium (green).

bioRxiv preprint doi: https://doi.org/10.1101/2025.04.28.651085; this version posted May 2, 2025. The copyright holder for this preprint (which was not certified by peer review) is the author/funder, who has granted bioRxiv a license to display the preprint in perpetuity. It is made available under aCC-BY 4.0 International license.

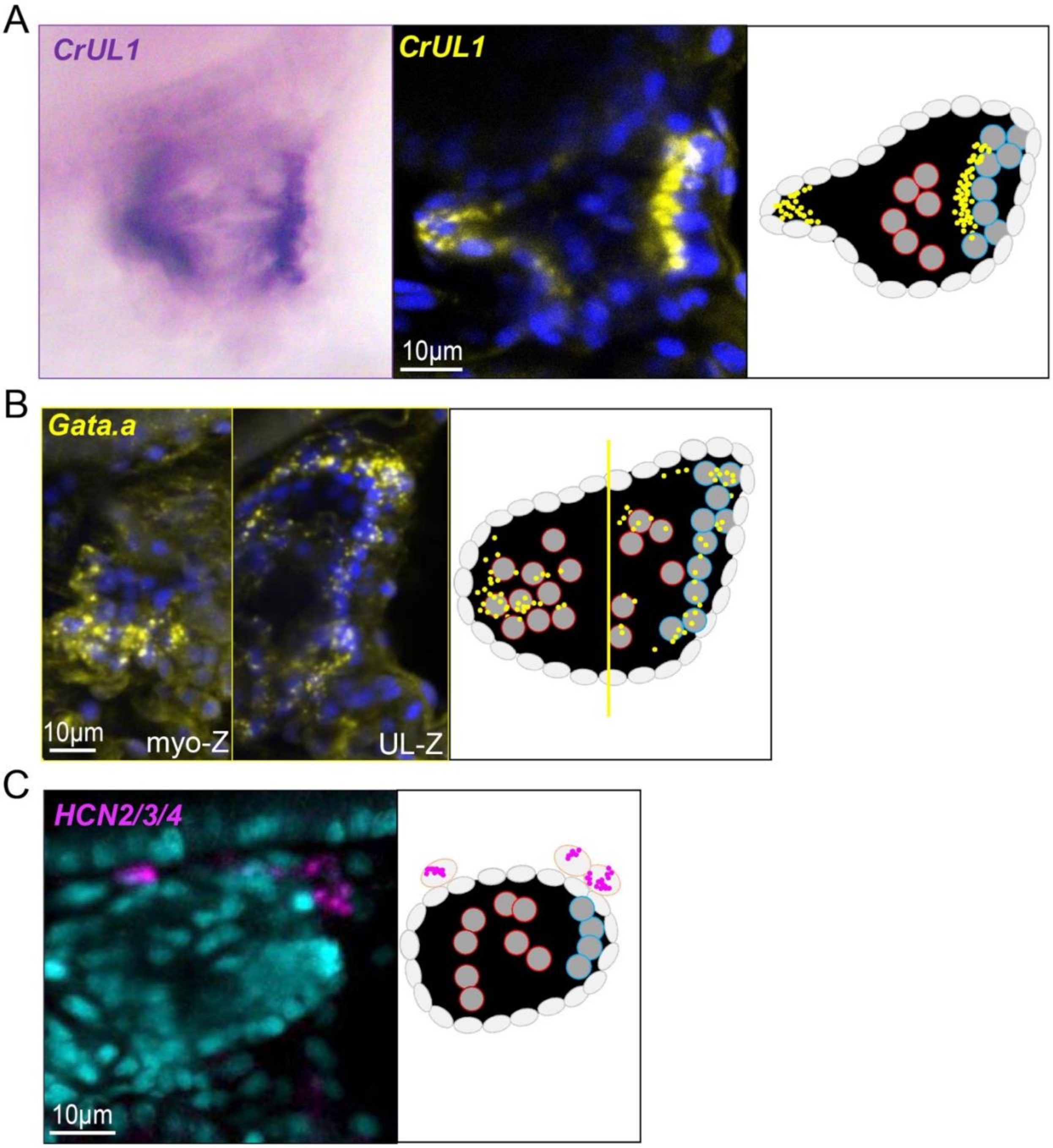

S7 Figure. Expression patterns of marker genes associated with the presumptive UL and cardiac neural-like clusters. (A) *CrUL1* expression in a D5 heart. Colorimetric in situ hybridization (left), FISH (middle), and cartoon schematic (right). **(B)** *Gata.a* expression in a D8 heart. FISH (left) and cartoon (right). Note that in the micrograph a Z-plane containing

bioRxiv preprint doi: https://doi.org/10.1101/2025.04.28.651085; this version posted May 2, 2025. The copyright holder for this preprint (which was not certified by peer review) is the author/funder, who has granted bioRxiv a license to display the preprint in perpetuity. It is made available under aCC-BY 4.0 International license.

myocardial cells in the anterior region of the heart is shown on the left while the Z-plane containing the UL in the posterior region of the heart is shown on the right. **(C)** *HCN2/3/4* expression in a D5 heart. FISH (left) and cartoon depiction (right). Blue (A and B) or cyan (C) represents DAPI staining. Yellow (A and B) or magenta (C) represents probe detection for each transcript. All images shown anterior to the left and dorsal up.

bioRxiv preprint doi: https://doi.org/10.1101/2025.04.28.651085; this version posted May 2, 2025. The copyright holder for this preprint (which was not certified by peer review) is the author/funder, who has granted bioRxiv a license to display the preprint in perpetuity. It is made available under aCC-BY 4.0 International license.

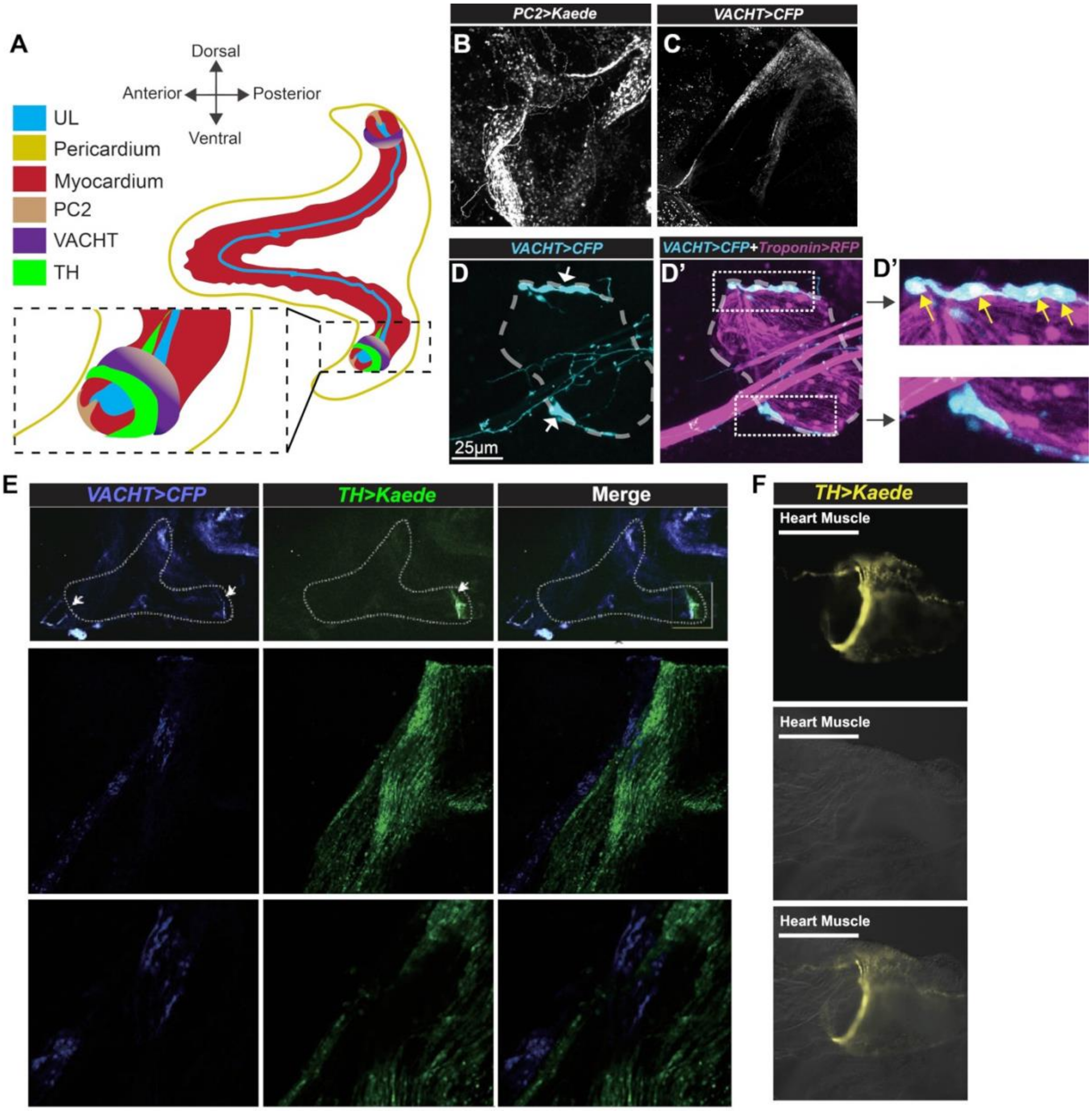

S8 Figure. Location of cardiac neural-like/pacemaker cells in the adult *C. robusta* heart.

(A) Summary diagram of observed localization patterns for PC2+, VACHT+, and TH+ neural-like cells in the distal plexus of the adult heart. Note the ventral-exclusive presence of TH (green). **(B)** *PC2>Kaede* expression. **(C)** *VACHT>CFP* expression. (**D,D', D''**) *VACHT>CFP* and *Troponin>RFP* expression in a young D30 adult as indicated. D'' shows magnified regions from D'. **(E)** *VACHT>CFP* and *TH>Kaede* double-labeled adult hearts. Left column is *VACHT>CFP* (blue), middle column is *TH>Kaede* (green), and right column is merged. Middle and bottom

bioRxiv preprint doi: https://doi.org/10.1101/2025.04.28.651085; this version posted May 2, 2025. The copyright holder for this preprint (which was not certified by peer review) is the author/funder, who has granted bioRxiv a license to display the preprint in perpetuity. It is made available under aCC-BY 4.0 International license.

rows correspond to enlarged areas of two different Z-planes of the boxed area in the top row. **(F)** *TH>Kaede* reporter expression at the distal end of the ventral plexus. Top panel is Kaede fluorescence, middle panel is brightfield, bottom panel is merged. Line in **F** indicates where the myocardial tube ends relative to the TH+ neural-like ring.

bioRxiv preprint doi: https://doi.org/10.1101/2025.04.28.651085; this version posted May 2, 2025. The copyright holder for this preprint (which was not certified by peer review) is the author/funder, who has granted bioRxiv a license to display the preprint in perpetuity. It is made available under aCC-BY 4.0 International license.

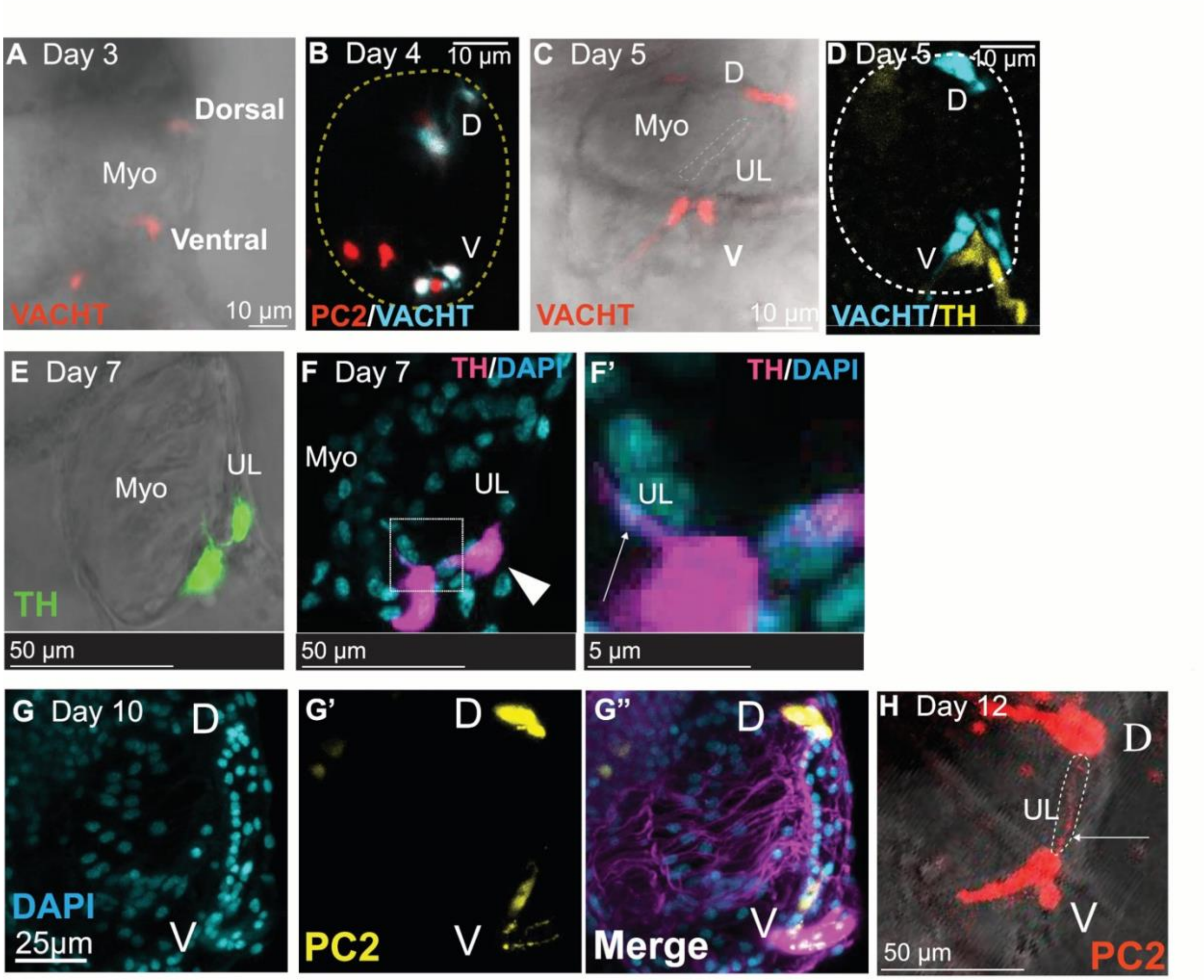

S9 Figure. Location of cardiac neural-like/pacemaker cells in the juvenile heart. (A) *VACHT>CFP* (red) in live D3 juvenile heart. **(B)** *PC2>Kaede* (red) and *VACHT>CFP* (blue) in a fixed D4 juvenile heart, outlined in yellow. **(C)** *VACHT>CFP* (red) in live D5 juvenile heart. **(D)** *VACHT>CFP* (blue) and *TH>Kaede* (yellow) in a fixed D5 juvenile heart, outlined in white. **(E)** *TH>Kaede* in a live D7 juvenile heart. **(F)** *TH>Kaede* (magenta) in a fixed juvenile heart. DAPI staining in cyan. F' shows a single Z-plane from F, note the overlap in magenta and cyan that appears to be associated with a UL cell (arrow). **(G)** DAPI stained nuclei of a D10 transgenic *PC2>Kaede* juvenile heart. G' shows *PC2>Kaede* expression. G'' shows merged view. Note *PC2> Kaede* expression at the dorsal and ventral ends of the UL. **(H)** *PC2>Kaede* expression (red) in a D12 juvenile heart. Note staining at the dorsal and ventral ends of the UL as well as

bioRxiv preprint doi: https://doi.org/10.1101/2025.04.28.651085; this version posted May 2, 2025. The copyright holder for this preprint (which was not certified by peer review) is the author/funder, who has granted bioRxiv a license to display the preprint in perpetuity. It is made available under aCC-BY 4.0 International license.

expression along the UL (arrow and dotted line). In A, C, E, and H, fluorescence merged with brightfield.

bioRxiv preprint doi: https://doi.org/10.1101/2025.04.28.651085; this version posted May 2, 2025. The copyright holder for this preprint (which was not certified by peer review) is the author/funder, who has granted bioRxiv a license to display the preprint in perpetuity. It is made available under aCC-BY 4.0 International license.

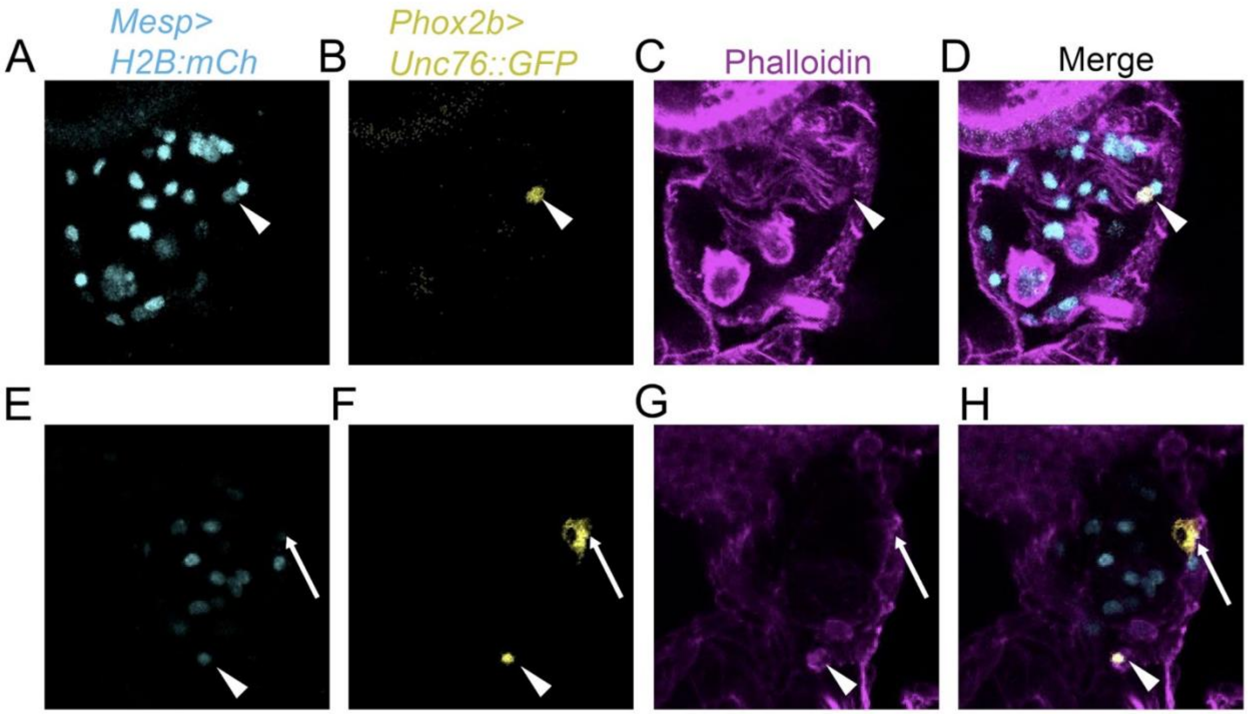

S10 Figure. Identification of presumptive pacemaker cells that co-express *Phox2b* and *Mesp* reporters. (A-D) Juvenile *MESP>H2B:mCh* (cyan) *Phox2b>Unc76::GFP* (yellow) heart stained with phalloidin (magenta). The only Phox2b+ cell shown in this substack is also Mesp+ (arrowhead). **(E-H)** Another juvenile *MESP>H2B:mCh* (cyan) *Phox2b>Unc76::GFP* (yellow) heart stained with phalloidin (magenta). Of the two Phox2b+ cells shown in this substack, one is Mesp- (arrow) while the other is Mesp+ (arrowhead). Note that the morphology of the Mesp+ cells is distinct from that of the Mesp- cell. A total of 9 transgenic hearts were examined and these are the only two double positive cells observed among a total of 37 Phox2b+ cells that were detected in these hearts.

bioRxiv preprint doi: https://doi.org/10.1101/2025.04.28.651085; this version posted May 2, 2025. The copyright holder for this preprint (which was not certified by peer review) is the author/funder, who has granted bioRxiv a license to display the preprint in perpetuity. It is made available under aCC-BY 4.0 International license.

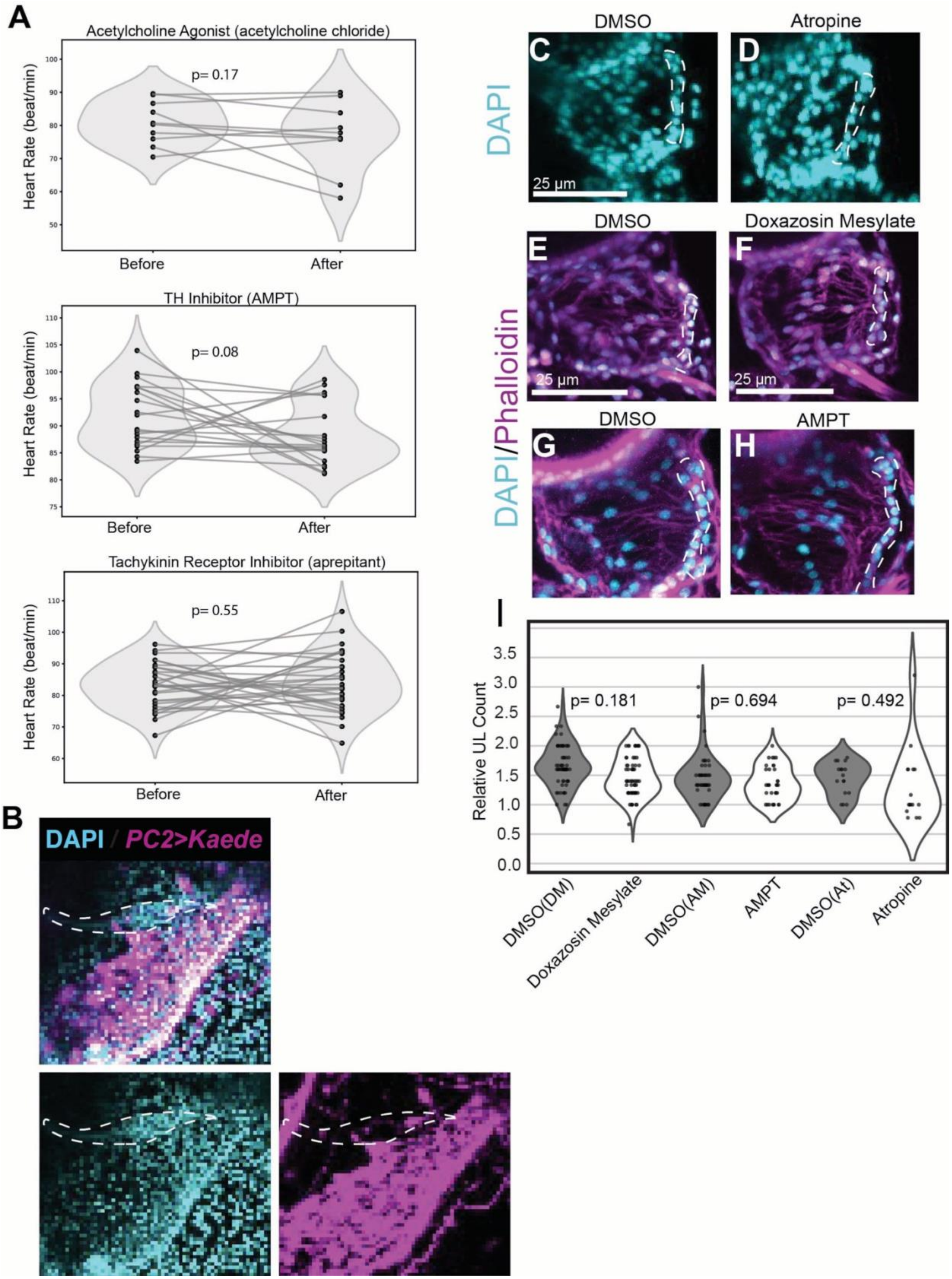

827

bioRxiv preprint doi: https://doi.org/10.1101/2025.04.28.651085; this version posted May 2, 2025. The copyright holder for this preprint (which was not certified by peer review) is the author/funder, who has granted bioRxiv a license to display the preprint in perpetuity. It is made available under aCC-BY 4.0 International license.

S11 Figure. The impact of neural signaling modulators on heart rate and UL cell numbers. (A) Violin plots of recorded heart rates in response to acetylcholine chloride (top), AMPT (middle), and aprepitant (bottom). **(B)** *PC2>Kaede* (magenta) labels cells that are interspersed with densely clustered DAPI stained nuclei (cyan) within the distal UL (outlined in white) in an adult heart. **(C)** D7 control heart. **(D)** D7 heart treated with Atropine. **(E)** D7 control heart. **(F)** D7 heart treated with doxazosin mesylate. **(G)** D7 control heart. **(H)** D7 heart treated with AMPT. **(I)** Violin plot of normalized UL counts per treatment. Counts normalized to the DMSO control for each trial. In C-H, cyan represents DAPI stained nuclei. Images in E-H also show phalloidin staining (magenta). For I, a t-test was performed on normalized data, averaged across trials. Doxazosin Mesylate: N= 60 control and 58 experimental samples, 2 trials. Atropine: N=13 control and 14 experimental samples, 2 trials. AMPT: N= 46 control, 33 experimental samples, 2 trials.

bioRxiv preprint doi: https://doi.org/10.1101/2025.04.28.651085; this version posted May 2, 2025. The copyright holder for this preprint (which was not certified by peer review) is the author/funder, who has granted bioRxiv a license to display the preprint in perpetuity. It is made available under aCC-BY 4.0 International license.

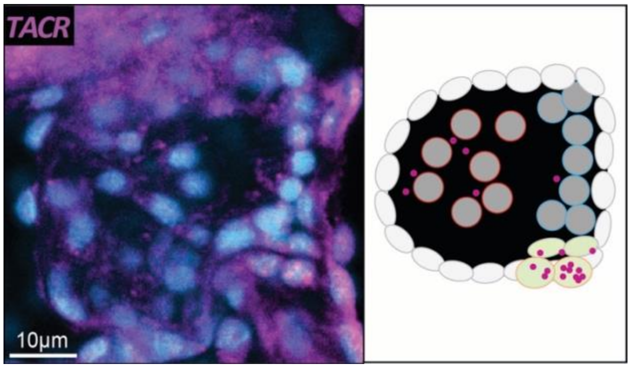

S12 Figure. Fluorescent in situ hybridization of *TACR* expression. Blue represents DAPI and magenta displays fluorescent probe detection (left). Cartoon model of expression pattern (right), UL cells outlined in blue, myocardial cells outlined in red, green cells represent presumptive neural-like/pacemaker cells.

bioRxiv preprint doi: https://doi.org/10.1101/2025.04.28.651085; this version posted May 2, 2025. The copyright holder for this preprint (which was not certified by peer review) is the author/funder, who has granted bioRxiv a license to display the preprint in perpetuity. It is made available under aCC-BY 4.0 International license.

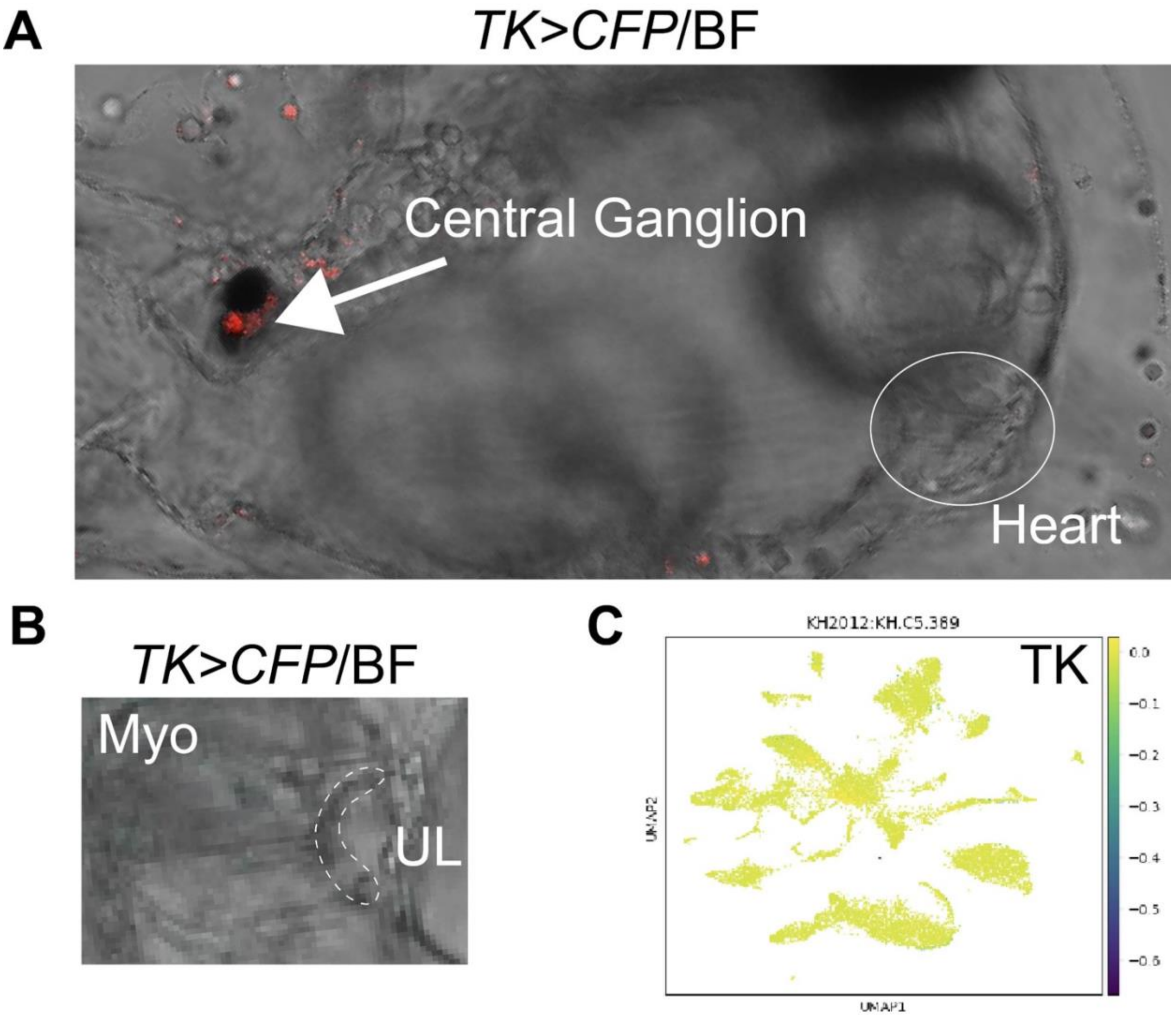

S13 Figure. Tachykinin reporter and scRNA-seq analysis confirms lack of *Cr-TK* expression in the heart. (A) *TK>CFP* reporter electroporation expression in the central ganglion (red). (B) Magnified region of the heart. Note absence of reporter expression. (C) UMAP showing *Cr-TK* expression levels, note absence of expression.

bioRxiv preprint doi: https://doi.org/10.1101/2025.04.28.651085; this version posted May 2, 2025. The copyright holder for this preprint (which was not certified by peer review) is the author/funder, who has granted bioRxiv a license to display the preprint in perpetuity. It is made available under aCC-BY 4.0 International license.

S1 Movie. Control D10 juvenile exhibiting stereotypical peristalsis. Juvenile expressing *PC2>Cas9* and a GFP-targeting sgRNA.

S2 Movie. Experimental D10 juvenile exhibiting a pericardial bubble absent a prominent inner myocardial tube. Juvenile expressing *PC2>Cas9* and a pair of *TK*-targeting sgRNAs.

bioRxiv preprint doi: https://doi.org/10.1101/2025.04.28.651085; this version posted May 2, 2025. The copyright holder for this preprint (which was not certified by peer review) is the author/funder, who has granted bioRxiv a license to display the preprint in perpetuity. It is made available under aCC-BY 4.0 International license.

S3 Movie. Control D10 juvenile exhibiting stereotypical peristalsis. Juvenile expressing *Mesp>Cas9* and a GFP-targeting sgRNA.

S4 Movie. Experimental D10 juvenile showing dramatically disrupted myocardial tube along with abnormal peristalsis. Juvenile expressing *Mesp>Cas9* and a pair of *TACR*-targeting sgRNAs.
